# Optimization of Mixed-Culture Solid-State Fermentation and the Effect of Microencapsulation on the Antioxidant Activity of Cherry Blossom Extract

**DOI:** 10.3390/ijms27115077

**Published:** 2026-06-04

**Authors:** Xiao Zhang, Liuxin Shi, Bing Zhang, Minghui Xu

**Affiliations:** College of Light Industry and Food Engineering, Nanjing Forestry University, Nanjing 210037, China; 3231500679@njfu.edu.cn (X.Z.); bingzhang@njfu.edu.cn (B.Z.); 3168876459@njfu.edu.cn (M.X.)

**Keywords:** cherry blossom, mixed-culture solid-state fermentation, response surface methodology, microencapsulation, antioxidant activity

## Abstract

Cherry blossom extract (CBE) is rich in natural antioxidants; however, the poor release of bound bioactive compounds and the instability of the extract itself limit its further application. This study aimed to improve the antioxidant activity and stability of CBE by combining mixed-culture solid-state fermentation with microencapsulation. Using total phenolic content (TPC) as the primary response variable, we optimized the solid-state fermentation conditions for a mixed culture of *Bacillus subtilis* and *Monascus purpureus* via single-factor experiments combined with the Box–Behnken response surface method. Extracts prepared under these optimal conditions were then microencapsulated using a gelatin–chitosan co-precipitation method, and their antioxidant activity and in vitro release behavior were evaluated. The optimal fermentation conditions were an inoculation amount of 10.5%, an inoculation proportion of 1.3:1 (*w*/*w*), and a fermentation time of 7.3 days, producing a total phenolic content (TPC) of 38.49 ± 0.41 mg gallic acid equivalent (GAE)/g dry weight (DW). After 150 h, the micro-capsules had released 49.6% of their bioactive contents, with 50.4% remaining encapsulated. Collectively, these findings demonstrate that the mixed-culture fermentation extract exhibited higher antioxidant activity than both the single-strain extract and the non-fermented control, thereby overcoming key application bottlenecks of CBE.

## 1. Introduction

During complex aerobic metabolic processes in living organisms, leakage of electrons from the mitochondrial respiratory chain, as well as exposure to external environmental stressors such as ultraviolet radiation and pollution, can induce the production of large amounts of reactive oxygen species (ROS) [1]. When the rate at which ROS are produced in the body exceeds the clearance capacity of the endogenous antioxidant defense system, a severe oxidative stress response is triggered [2,3]. Chronic oxidative stress can lead to the lipid peroxidation of cell membranes, the denaturation and misfolding of proteins, and DNA strand breaks [3,4]. These processes can cause tissue aging and contribute to the pathological development of cardiovascular diseases, neurodegenerative diseases, diabetes, and various malignant tumors [3,4,5]. Consequently, identifying safe, effective and non-toxic natural antioxidants to replace certain synthetic antioxidants (e.g., BHT and BHA), which pose potential health risks, has become a key area of research in food science, cosmetic science and modern preventive medicine [6,7,8].

Cherry blossoms hold great ornamental value in landscaping and their petals have been proven to contain a variety of bioactive compounds, making them a natural treasure trove in both traditional medicine and modern phytochemical research [9,10,11]. Studies involving extensive chromatographic separation and structural identification have shown that cherry blossom petals contain high levels of flavonoids (e.g., rutin and quercetin and their glycoside derivatives), anthocyanins, polyphenolic acids (e.g., caffeic acid, p-coumaric acid and chlorogenic acid) and various carotenoids [10,11,12]. These active components commonly feature molecular structures with multiple conjugated phenolic hydroxyl systems that act as excellent hydrogen or singlet electron donors, neutralizing highly reactive oxidants such as superoxide anion radicals and hydroxyl radicals [6,7,8,10]. This interrupts the radical chain reaction of lipid peroxidation, demonstrating exceptional antioxidant efficacy in vitro and in vivo [10,11,13]. Furthermore, CBE has been shown to effectively inhibit tyrosinase activity and reduce melanin production while significantly downregulating the expression of inflammatory genes. CBE therefore demonstrates significant commercial potential in cosmetics and pharmaceuticals, including anti-aging, anti-inflammatory, soothing, and skin-brightening and repair products [9,14,15,16].

However, in actual extraction processes, there is a significant discrepancy between the extraction efficiency of natural antioxidants from cherry blossom petals and their theoretical content [17,18,19,20]. Studies in plant cytology have shown that phenolic compounds in plants do not exist entirely in a free state within vacuoles [17,21]. In fact, as much as 40–60% of phenolic acids and flavonoids form strong covalent cross-links with cellulose, hemicellulose, pectin and lignin in the cell wall via ester, ether or glycosidic bonds [17,18,21]. This forms insoluble bound polyphenols that are extremely difficult to dissolve in conventional solvents [17,21]. Traditional physical and chemical methods such as solvent extraction, ultrasonic-assisted extraction or microwave-assisted extraction often only extract free polyphenols present in the cytoplasm; they are powerless against antioxidant compounds encapsulated within or covalently bound to dense cell walls. Furthermore, excessive heating at high temperatures or treatment with strong acids and bases not only consumes enormous amounts of energy and is highly prone to causing environmental pollution, but also leads to the irreversible oxidative degradation of heat-sensitive anthocyanins and flavonoids, severely impairing their biological activity [19,20].

In recent years, solid-state fermentation has been recognized as an effective strategy for improving the release of bioactive plant compounds. It can degrade plant tissue structures using enzymes such as cellulases and xylanases, and promote the restructuring of phenolic compounds and enhance antioxidant capacity through microbial biotransformation [22,23,24,25,26,27,28]. However, although fermentation extracts are highly bioactive, they are sensitive to light, heat and oxygen and prone to degradation during processing and storage. Microencapsulation, particularly systems based on the co-aggregation of natural polymers, is widely recognized as a method of improving the stability of active ingredients and achieving a sustained-release profile [29,30,31,32,33,34,35,36]. Nevertheless, previous studies on cherry blossom extracts have mainly focused on compositional identification, extraction or bioactivity evaluation, whereas the release of bound phenolics and the subsequent stabilization of fermentation-enhanced extracts have not been sufficiently addressed. In particular, the combination of mixed-culture solid-state fermentation with microencapsulation has rarely been explored for cherry blossom resources.

Based on this, the present study employed cherry blossoms as the raw material for establishing a mixed-culture, solid-state fermentation system involving *Bacillus subtilis* and *Monascus purpureus*. This system was combined with gelatin–chitosan co-polymer microencapsulation technology with the aim of developing a technical system that would enable the efficient release and stabilization of active components from cherry blossoms. The results suggest that mixed microbial fermentation promotes the release of phenolic compounds and enhances antioxidant capacity, while microencapsulation facilitates the sustained release and retention of the components’ functionality. This provides experimental evidence for the effective utilization of cherry blossom resources. The novelty of this study therefore lies in constructing an integrated release-stabilization strategy, in which *Bacillus subtilis*–*Monascus purpureus* co-fermentation is used to enhance phenolic release and gelatin–chitosan microencapsulation is used to protect and sustain the antioxidant functionality of the extract.

## 2. Results and Discussion

### 2.1. Changes in Enzyme Activity of Different Strains During Monoculture Fermentation

**Figure 1:**
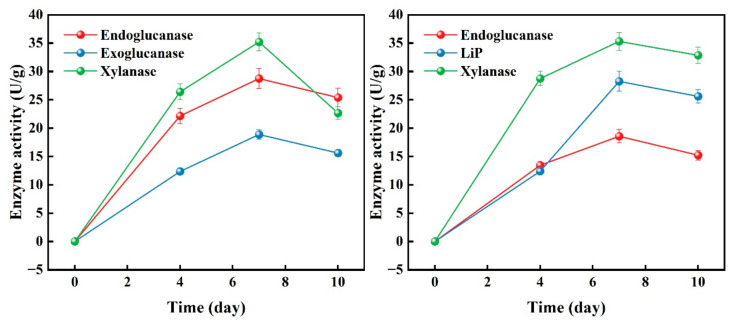
Changes in enzyme activity during fermentation. The image on the left shows *Monascus purpureus*, and the image on the right shows *Bacillus subtilis*.

As shown in Figure 1,the results indicate that the activities of key degrading enzymes in both single-strain fermentation systems underwent significant dynamic changes throughout the fermentation process, although the enzyme profiles and the extent of these changes differed among the various strains. In the *Monascus purpureus* fermentation system, endoglucanase activity increased from 0 U/g to 22.15 U/g by day 4, peaked at 28.74 U/g on day 7, and decreased slightly to 25.38 U/g by day 10; exo-glucanase activity also followed an initial rise followed by a decline, with values of 12.35 U/g, 18.88 U/g, and 15.60 U/g at days 4, 7, and 10, respectively, with the highest value observed on day 7. Xylanase activity showed the most significant increase, rising from 0 U/g to 26.40 U/g at 4 days, reaching a peak of 35.20 U/g at 7 days, and subsequently decreasing to 22.65 U/g at 10 days. In the *Monascus purpureus* fermentation group, no significant activity of lignin peroxidase (LiP) was detected throughout the entire fermentation cycle. These results indicate that the degradation of cherry cell walls by *Monascus purpureus* primarily relies on the synergistic action of cellulase and hemicellulase systems. In particular, the high expression of xylanase and endoglucanase during the middle of the fermentation period facilitates the disruption of the hemicellulose backbone and the amorphous regions of cellulose, thereby promoting the release of bound phenolic and flavonoid compounds.

In contrast, the *Bacillus subtilis* fermentation system exhibited a different distribution of enzyme activity. Its endoglucanase activity was 13.45 U/g, 18.56 U/g, and 15.22 U/g at 4, 7, and 10 days, respectively, also peaking at 7 days, but the overall level was lower than that of the *Monascus purpureus* group; no significant exoglucanase activity was detected throughout the fermentation process. Xylanase, however, remained at a consistently high level, reaching 28.75 U/g at 4 days, further increasing to 35.30 U/g at 7 days, and still maintaining a high level of 32.85 U/g at 10 days, indicating that *Bacillus subtilis* possesses a strong and sustained ability to degrade hemicellulose components. It is worth noting that the *Bacillus subtilis* group exhibited relatively significant lignin peroxidase activity, with values of 12.40 U/g, 28.25 U/g, and 25.60 U/g at 4, 7, and 10 days, respectively, reaching a peak at 7 days. The presence of LiP activity indicates that *Bacillus subtilis* not only acts on the polysaccharide backbone but also oxidatively degrades lignin and aromatic cross-linked structures, further weakening the cell wall barrier and hydrophobic barrier layer of cherry blossom tissue, thereby facilitating the release of antioxidant active components embedded within the cell wall complex. A comprehensive comparison reveals that *Monascus purpureus* exhibits greater advantages in the cellulase system, particularly with higher endoglucanase and exoglucanase activities, indicating its greater proficiency in directly cleaving the cellulose backbone, whereas *Bacillus subtilis* stands out in xylanase and LiP activities, suggesting its superiority in hemicellulose degradation and lignin oxidation for cell wall disruption. The activity of most key enzymes in both strains peaked on day 7, indicating that this period represents the most active phase for substrate degradation and the release of bioactive compounds. By day 10, some enzyme activities had declined, possibly due to substrate depletion, accumulation of metabolic byproducts, and the transition of bacterial growth from the logarithmic phase to the stationary phase. These results indicate that different bacterial species exhibit distinct enzyme-producing roles during single-strain fermentation: *Monascus purpureus* focuses on the hydrolysis of the cellulose backbone, while *Bacillus subtilis* focuses on the disruption of hemicellulose and lignin barriers. Together, they demonstrate that microbial fermentation can deconstruct the tissue structure of cherry blossoms in layers via extracellular enzyme systems, providing an enzymatic basis for the subsequent intensification of microstructural disruption and the enhancement of antioxidant activity in the extract. Mechanistically, *Monascus purpureus* tends to break down the polysaccharide framework, while *Bacillus subtilis* tends to weaken the lignin and hydrophobic barriers. Consequently, this provides a theoretical foundation for further exploration of synergistic effects through mixed-microbial fermentation.

### 2.2. Results of Single-Factor Experiments on Mixed-Culture Solid-State Fermentation

Based on previous exploratory studies of single-strain fermentation, it is evident that the metabolic pathways of a single strain are often insufficient to optimize the release of highly complex plant natural products [22,23,24,25,26,27,28]. Although *Monascus purpureus* has strong enzyme-producing capabilities, its mycelium growth is slow in the early stages of fermentation [28,37,38]. In contrast, *Bacillus subtilis* produces cellulase slightly less efficiently, but its growth cycle is extremely short [39,40]. This enables it to rapidly alter the microenvironment of the solid-state substrate and reduce the rigidity of the plant cell membrane lipid bilayer [22,23,39,40]. Consequently, it can promote the elution of intracellular polyphenols and fat-soluble components synergistically. This chapter therefore proposes establishing a mixed-culture fermentation system using a dual-strain co-culture of *B. subtilis* and *M. purpureus* to maximize the extraction yield of antioxidants from cherry blossoms through interspecies metabolic interactions and enzymatic complementarity [22,23,24,25,26,27,28,37,38,39,40].

#### 2.2.1. The Effect of Inoculation Amount on Total Phenolic Content

**Figure 2:**
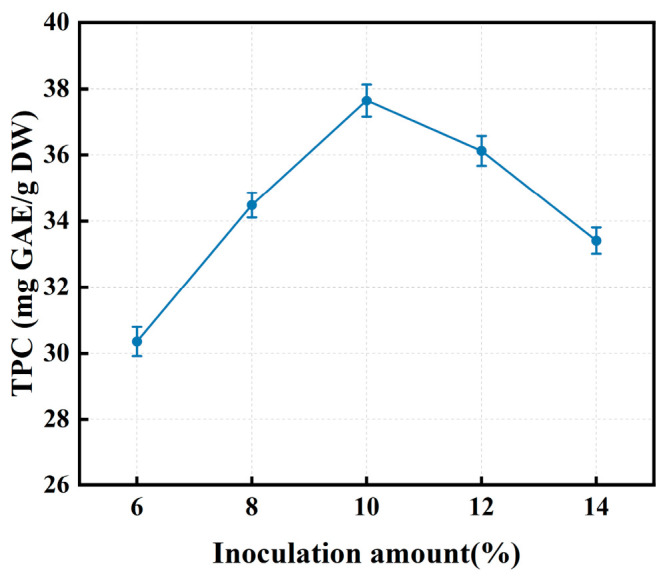
The effect of inoculation amount.

When the inoculation proportion and fermentation time were both fixed at 1:1 and 7 days respectively, it was found that the inoculation amount significantly affected the total phenolic content (TPC) of the mixed-culture fermentation extract of cherry blossoms. As shown in Figure 2, TPC rose significantly from 30.36 ± 0.44 mg GAE/g DW to 37.64 ± 0.49 mg GAE/g DW as the inoculation amount increased from 6% to 10%. However, when the inoculation amount was increased further to 12% and 14%, TPC decreased to 36.12 ± 0.45 mg GAE/g DW and 33.41 ± 0.40 mg GAE/g DW respectively, indicating an initial increase followed by a decrease. These results suggest that moderate increases in inoculation amount facilitate the rapid colonization of both strains and the establishment of a synergistic enzyme production system. This promotes plant cell wall degradation and the release of bound phenolics. However, excessively high inoculation amount may intensify substrate competition, leading to localized metabolic stress and the accumulation of metabolites. This is detrimental to the subsequent accumulation of active components. Based on these results, the inoculation amount range was set at 8–12% for subsequent response surface optimization.

#### 2.2.2. The Effect of the Inoculation Proportion on Total Phenol Content

**Figure 3:**
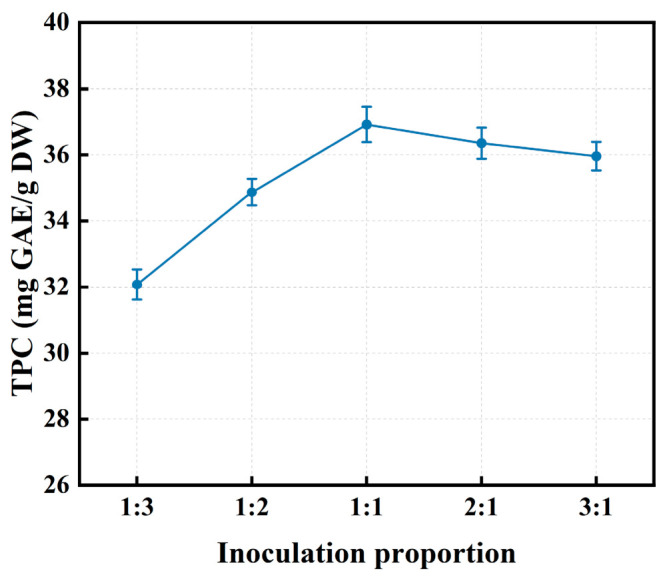
The effect of inoculation proportion.

When the total inoculation amount was set to 10% and the fermentation time to 7 days, the total plate count (TPC) also exhibited an initial increase followed by a decrease as the ratio of *Bacillus subtilis* to *Monascus purpureus* inoculation was varied. As shown in Figure 3, the TPC values were 32.08 ± 0.46, 34.87 ± 0.41, 36.91 ± 0.53, 36.35 ± 0.47 and 35.96 ± 0.43 mg GAE/g DW, respectively, when the inoculation proportions were 1:3, 1:2, 1:1, 2:1 and 3:1, with the 1:1 treatment group reaching the highest value. These results suggest that the two strains exhibit significant enzymatic complementarity in the mixed microbial system. When the proportion of *Monascus purpureus* is high, initial colonization is slow, hindering rapid substrate surface degradation. Conversely, when the proportion of *Bacillus subtilis* is high, initial metabolism is rapid, but deep penetration and sustained cell wall lysis are insufficient in the later stages. Overall, balanced inoculation is more conducive to establishing a stable synergistic degradation environment. To facilitate model development, this study converted the inoculation proportion to the volume fraction of *Bacillus subtilis*, selecting 0.33–0.67 as the subsequent response surface optimization range.

#### 2.2.3. The Effect of Fermentation Time on Total Phenolic Content

**Figure 4:**
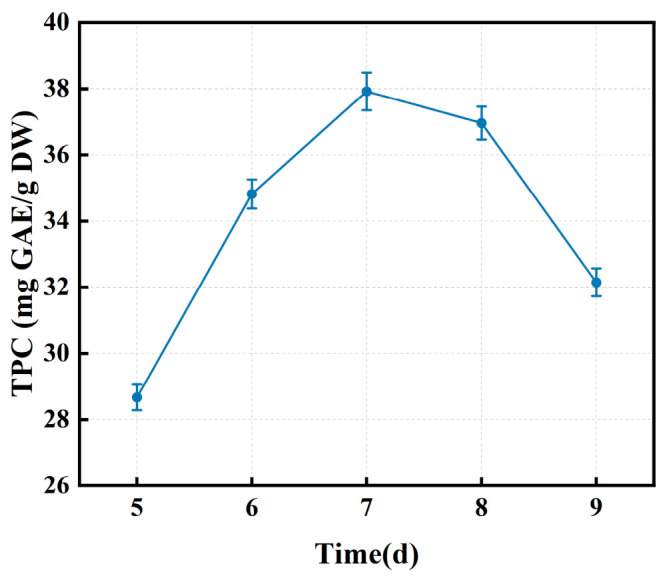
The effect of fermentation time.

When the total inoculation amount was fixed at 10% and the inoculation proportion at 1:1, TPC exhibited a typical single-peak pattern as the fermentation time increased. As shown in Figure 4, extending the fermentation time from 5 to 7 days resulted in a significant increase in TPC from 28.67 ± 0.39 mg GAE/g DW to 37.92 ± 0.57 mg GAE/g DW. Further extending the fermentation time to 8 and 9 days caused a decrease in TPC to 36.96 ± 0.50 mg GAE/g DW and 32.15 ± 0.42 mg GAE/g DW, respectively. These results suggest that the 5–7 day period is the stage at which synergistic enzyme production, continuous cell wall disruption and phenolic conversion are most active for both strains. However, excessive fermentation may result in the further metabolic utilization of released phenolics, or oxidative polymerization and degradation under prolonged exposure conditions. Based on the results of the single-factor analysis, this study selected 7 days as the central point and 6–8 days as the response surface optimization range.

### 2.3. Response Surface Optimization of Mixed-Culture Fermentation Processes

#### 2.3.1. Regression Model Specification and Analysis of Variance

Based on the results of the single-factor experiments, a three-factor, three-level quadratic polynomial regression model was constructed using the Box–Behnken design, with inoculation amount (A), *Bacillus subtilis* volume fraction (B), and fermentation time (C) as independent variables and TPC as the response variable, as shown in Table 1. As shown by the results of the 17 experimental groups in Table 2, the TPC for the central point treatments ranged between 37.84 and 38.54 mg GAE/g DW, indicating good experimental reproducibility. The following regression equation was obtained using Design-Expert 13.0 for fitting [41,42]:

Y TPC = 38.20 + 0.7763A + 0.6213B + 1.39C + 0.5525AB + 0.1550AC − 0.1050BC − 1.90A^2^ − 1.13B^2^ − 2.22C^2^ (1)

The regression coefficients indicate that the linear term coefficients for A, B and C are all positive, whereas the quadratic term coefficients are all negative. This suggests that all three factors have a positive effect on TPC within the range of analysis, but there is a distinct local maximum in the response values.

As shown in Table 3, the results of the analysis of variance (ANOVA) suggest that the model is highly significant (F = 67.49, *p* < 0.0001), while the residual term is not (*p* = 0.4073). This indicates that the model fits the data well and is capable of making accurate predictions. All three main effects were significant: fermentation time (C) had the strongest effect (F = 133.85, *p* < 0.0001), followed by inoculation amount (A) (F = 41.59, *p* = 0.00035) and then inoculation proportion (B) (F = 26.64, *p* = 0.00131). The order of the main effects was therefore C > A > B.

Among the interaction terms, only AB was significant (*p* = 0.0141), while AC and BC were both insignificant. This suggests a certain synergistic effect between inoculation amount and inoculation proportion, while fermentation time primarily manifests as an independent main effect.

#### 2.3.2. Response Surface and Contour Analysis

**Figure 5:**
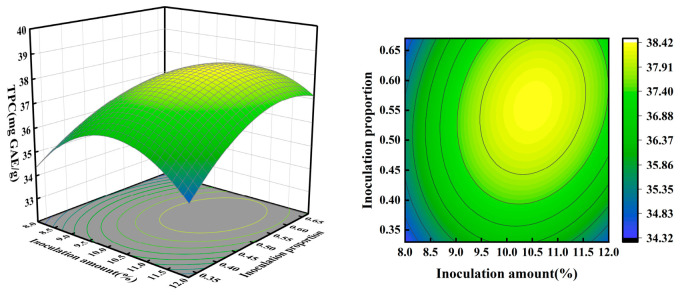
The interaction between inoculation amount and inoculation proportion.

Figure 5 illustrates the synergistic regulatory relationship between inoculation amount and inoculation proportion in regard to total phenolic content when the fermentation duration is kept at the central level. Combined with the results of the analysis of variance, it can be seen that the AB term reached a significant level, indicating that there is a true interaction between these two factors rather than a simple independent additive effect. Based on the positive coefficient of AB (+0.5525) in the regression equation, it can be further concluded that, within the examined range, moderately increasing the total inoculation amount while simultaneously increasing the proportion of *Bacillus subtilis* will have a positive synergistic effect on TPC. Consequently, the response surface in Figure 5 should exhibit a distinct upward trend, with contour lines that are more elliptical in shape compared to the other two figures, and peak regions concentrated around A ≈ 10–11% and B ≈ 0.50–0.58. This result indicates that increasing the inoculation amount alone or adjusting the bacterial inoculation proportion alone can only marginally enhance polyphenol release. However, when both factors are optimized within the optimal window, the colonization rates of both bacteria, substrate utilization, and the complementary effects of their extracellular enzyme systems are simultaneously enhanced, thereby more effectively disrupting the cell wall structure of cherry blossoms and promoting the release of bound phenolics. At the same time, since both the A^2^ and B^2^ terms are negative and highly significant, the surface curve does not continue to rise but instead reaches a distinct peak near the mid-to-high range; beyond this range, TPC actually decreases. This aligns with the patterns observed in the single-factor experiments, where “excessively high inoculation amount leads to intensified substrate competition and metabolic imbalance within the microbial community” and “an overly skewed ratio weakens the synergy between the two strains”.

**Figure 6:**
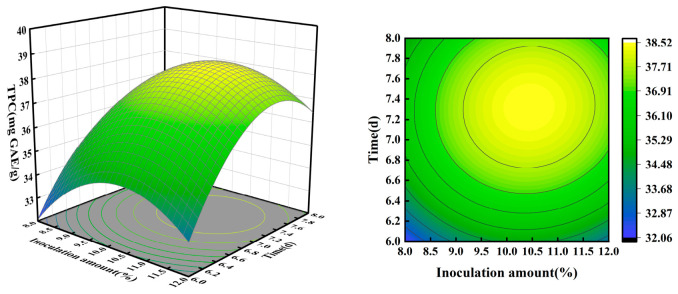
The interaction between inoculation amount and fermentation time.

Figure 6 shows the combined effect of total inoculation amount and fermentation time on TPC, assuming a constant inoculation proportion. While these two factors jointly determine the initial colonization scale and subsequent metabolic accumulation time in the mixed microbial system, the statistical results suggest that there is no significant coupling amplification effect between them. Therefore, while the response surface in Figure 6 shows a significant increase, this is primarily due to the main effect of fermentation time (C), rather than strong interaction between A and C. Since C has the highest F-value (133.85) and the largest first-order coefficient (+1.39), the slope along the C-axis should be considerably greater than the change along the A-axis. In other words, TPC is more sensitive to fermentation time than total inoculation amount. At the same time, both C^2^ and A^2^ are negative and significant. This indicates that the surface still exhibits a ‘rising-then-falling’ shape that opens downwards, with the peak region located near 10.5% for A and 7.3 days for C. It can therefore be concluded that a moderate inoculation amount ensures the rapid colonization of both strains. Meanwhile, a moderate extension of fermentation time promotes sustained extracellular enzyme activity, gradual cell wall degradation and the accumulation of bound phenolics. However, if fermentation time is too long, the released phenolics may undergo further metabolism by microorganisms or oxidative polymerization during prolonged exposure. This leads to a decline in TPC.

**Figure 7:**
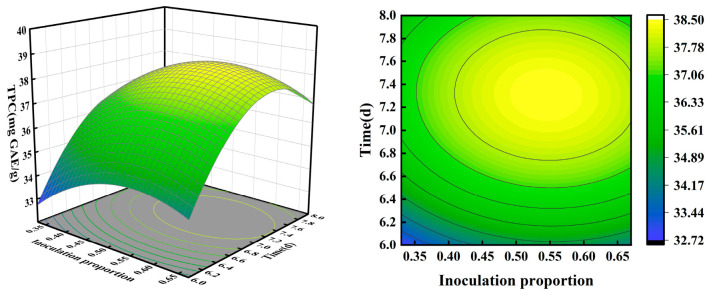
The interaction between inoculation proportion and fermentation time.

Figure 7 shows the combined effect of the inoculation proportion and fermentation time on total phenolic accumulation when the inoculation amount is set to its central value. Statistical analysis shows that the BC term is not significant and has the smallest regression coefficient absolute value (−0.1050), indicating virtually no significant synergistic or antagonistic interaction between these two factors. Specifically, extending the fermentation time from 6 days to approximately 7.3 days resulted in a significant increase in TPC; meanwhile, the effect of B was primarily manifested as a subtle adjustment to the peak position. This suggests that achieving optimal response values requires a moderately high level of B (around 0.50–0.56). In other words, fermentation time is the core variable determining the magnitude of TPC variation. This result aligns with the order of main effects (C > A > B) observed in other RSM studies, where ‘only a few interaction terms are significant, while the rest are primarily dominated by single factors’. Fermentation time directly determines the time window for enzymatic reactions and polyphenol release. Within a reasonable range, the inoculation proportion primarily affects system equilibrium, but is insufficient to reverse the overall trend caused by fermentation times that are too short or too long. Therefore, losses resulting from fermentation times that deviate from the optimal window cannot be compensated for by adjusting the proportion alone.

Overall, response surface analysis indicates that, although their mechanisms of action are not entirely consistent, all three factors exhibit a single-peak pattern of first increasing and then decreasing. The interaction between factors A and B was relatively pronounced, indicating that the total inoculation amount must be optimized in conjunction with the inoculation proportion to maximize the synergistic effect of the two strains. In contrast, fermentation time primarily determines the time window for enzymatic reactions, phenolic release, subsequent transformation, and degradation, making it a key factor in controlling the magnitude of changes in the response value. The results of the comprehensive model calculation indicate that the theoretically optimal inoculation amount is 10.53%, a *Bacillus subtilis* volume fraction of 0.555 (equivalent to a *Bacillus subtilis*:*Monascus purpureus* proportion of approximately 1.25:1) and a fermentation time of 7.32 days. Under these conditions, the predicted total phenolic content (TPC) is 38.62 mg GAE/g DW.

To ensure practical operability, the optimal process parameters were adjusted to a total Inoculation amount of 10.5%, an inoculation proportion of 1.3:1, and a fermentation time of 7.3 days, and three parallel experiments were conducted for validation. The results showed that the measured TPC of the sample was 38.49 ± 0.41 mg GAE/g DW, with a relative error of only 0.34% compared to the model prediction, indicating that the established response surface model possesses good predictive accuracy and practical guidance value. Compared with the single-strain system, mixed-strain solid-state fermentation not only increased the accumulation of total phenolics in cherry blossom petals but also provided an optimal process window for subsequent steady-state applications.

### 2.4. Evaluation of Physicochemical Properties and Biological Activity Following Microencapsulation

Although the optimized mixed-microbial solid-state fermentation process enhances the natural antioxidant activity of cherry blossom extract (CBE), the highly reactive phenolic hydroxyl groups in the polyphenolic compounds also contribute to their instability. These functional groups are susceptible to oxidative reactions when exposed to external factors such as oxygen, heat and light. This leads to irreversible browning and a decline in bioactivity, severely limiting their development and application in the field of natural products [29,30,31,32,33]. This chapter addresses this issue by using CBE obtained under optimal mixed-microbial fermentation conditions as the core material. Gelatin and chitosan, which possess good biocompatibility, are selected as composite wall materials. Co-precipitation technology is employed to construct a microencapsulation system, and the physicochemical properties are systematically characterized [29,30,31,32,33,34,35,36].

#### 2.4.1. Observation of the Surface Topography and Microstructure of Microcapsules

**Figure 8:**
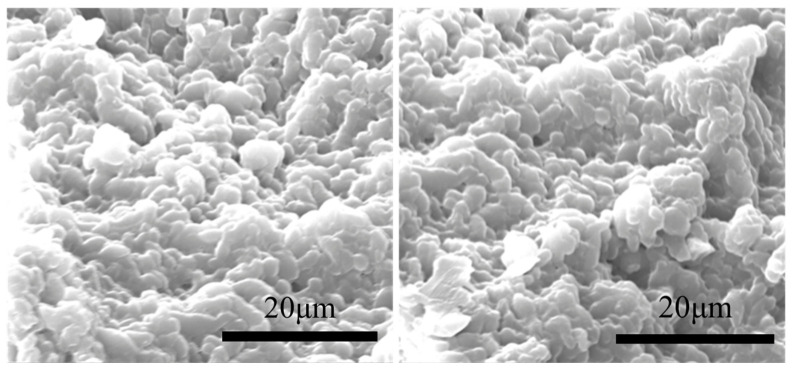
ESEM image of microcapsules containing CBE (CBE-M).

As shown in Figure 8, the microcapsules prepared in this study exhibit a primarily rough, wrinkled and agglomerated structure. Locally observable, nearly spherical protrusions are stacked on top of one another and the surface is dense overall. This indicates that, during the recoagulation process, a coating layer was successfully formed around the cherry blossom fermentation extract by gelatin and chitosan, which constructed a relatively stable three-dimensional network structure under the cross-linking action of glutaraldehyde [30,31,32,33,34,35,36]. During the subsequent freeze-drying and dehydration process, the wall materials shrink, causing the microcapsule particles to come into closer contact and adhere to one another. This results in a final morphology characterized by block-like aggregates and a wrinkled surface [30,34,35]. The absence of obvious collapse, rupture or large pore structures in the images indicates that the resulting microcapsules have good structural integrity. They can shield the core material from the effects of external oxygen, light and heat to some extent, which provides morphological evidence for the stable preservation and controlled release of the cherry blossom fermentation extract.

#### 2.4.2. Analysis of Fourier Transform Infrared Spectroscopy (FTIR)

**Figure 9:**
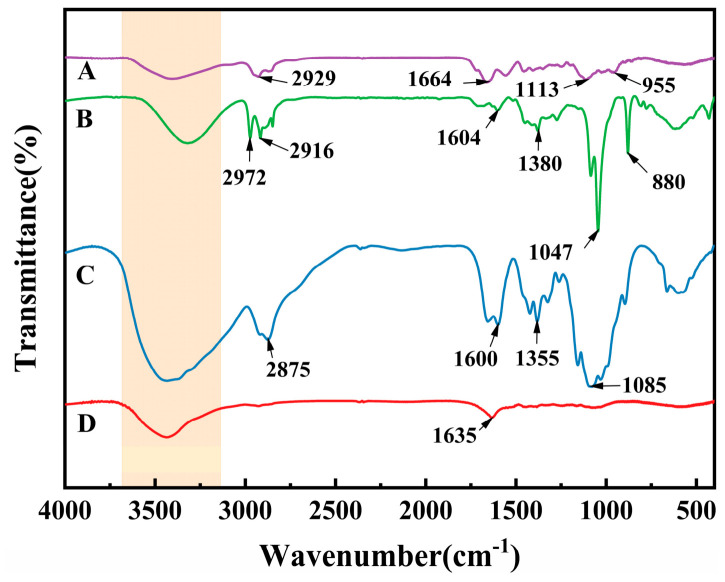
Fourier transform infrared (FTIR) spectra of different samples. A is microcapsule containing CBE (CBE-M), B represents cherry blossom extract (CBE), C is chitosan, and D is gelatin.

As shown in Figure 9, D (gelatin) exhibits a broad peak near 3400 cm^−1^, which can be attributed to N–H/O–H stretching vibrations; an amide I band appears near 1635 cm^−1^, reflecting the C=O stretching vibrations in the protein backbone, which is a typical infrared feature of gelatin. C (chitosan) also exhibits a broad and intense absorption band near 3400 cm^−1^, corresponding to the overlapping vibrations of –OH and –NH_2_; C–H stretching vibrations are visible near 2875 cm^−1^, amide-related absorption is visible near 1600 cm^−1^, and –OH stretching vibrations are visible near 1355 cm^−1^; the strong absorption in the 1085 cm^−1^ region is associated with C–O–C and C–O vibrations in the polysaccharide backbone, which is also an important characteristic of chitosan [33,36].

The characteristic peaks of B (CBE) are indicative of the core material’s chemical composition. The absorption peaks at 2972 and 2916 cm^−1^ correspond to asymmetric or symmetric stretching vibrations of –CH_3_/–CH_2_ in aliphatic chains. This indicates the presence of alkyl chains or lipid-soluble components in the extract [10,11,12]. The peak at 1604 cm^−1^ corresponds to C=C vibrations in the aromatic ring skeleton. The peak at 1380 cm^−1^ corresponds to –CH_3_ bending vibrations or vibrations associated with phenolic hydroxyl groups. The peak at 880 cm^−1^ lies in the fingerprint region and is more likely related to out-of-plane C–H bending vibrations in the aromatic ring or partially substituted aromatic ring structures. As the literature on cherry blossom extracts generally reports a high content of polyphenolic compounds, such as phenolic acids and flavonoids, it is reasonable for Sample B to exhibit distinct absorption peaks in the 1604/1380/880 cm^−1^ region [9,10,11,12].

Compared with B (CBE), A (CBE-M) still exhibits a broad peak near 3400 cm^−1^, but the peak is flatter and the bandwidth is wider. This indicates significant overlap and interaction between the –OH/–NH groups in the microcapsule wall material and the oxygen-containing groups in the core material [30,36]. At the same time, several characteristic absorption peaks in B are identifiable in A, but they are significantly less intense and overlap with the absorption bands of gelatin and chitosan. This suggests that the microcapsule spectrum reflects not only signals from the shell material, but also characteristic absorptions from the core material. However, since the core material is encapsulated within the shell, its apparent concentration in the system decreases and its vibrations are shielded and restricted by the shell. This results in the characteristic peaks appearing weakened, broadened or partially masked [30,34,35,36].

Furthermore, the A spectrum shows no new absorption bands that are sufficiently intense or distinctly different from those of the original wall or core materials. This suggests that the encapsulation process primarily involved re-aggregation and intermolecular interactions rather than significant new chemical reactions involving the cherry blossom extract. Microcapsule formation primarily relies on polyelectrolyte complexation between gelatin and chitosan, hydrogen bonding and subsequent curing to provide structural stability [29,30,31,32,33,34,35,36]. Meanwhile, the original major functional groups of the core material were not significantly disrupted after encapsulation, which is generally beneficial for maintaining the biological activity of the cherry blossom extract [29,30,31,32,33].

#### 2.4.3. Analysis of the Thermal Stability of Microcapsules

**Figure 10:**
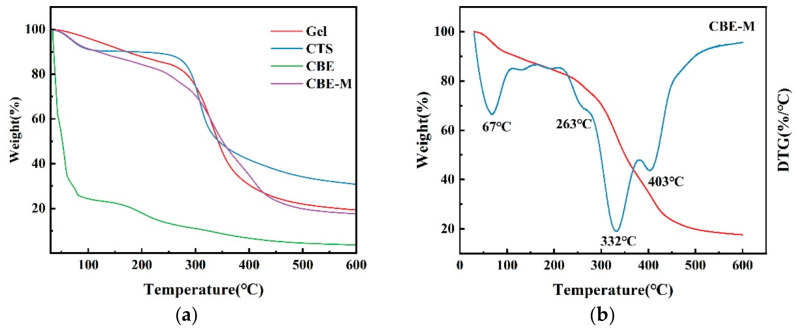
(**a**) TGA curves of gelatin (Gel), chitosan (CTS), cherry blossom extract (CBE) and microcapsule containing CBE (CBE-M); (**b**) DTG curves of CBE-M.

A comprehensive analysis of the TG and DTG curves (Figure 10) shows that the core material of the cherry blossom extract (CBE) loses a significant amount of weight even at low temperatures, indicating poor thermal stability. In contrast, the thermal decomposition process of the microcapsule sample (CBE-M) is significantly delayed after encapsulation via the gelatin–chitosan co-precipitation method, resulting in a more gradual, multi-stage weight loss profile [29,30,31,32,33,34,35,36]. The DTG curve shows characteristic peaks at 67 °C, 263 °C, 332 °C and 403 °C. These correspond to the volatilization of low-boiling-point components, initial structural cleavage, the rapid thermal decomposition of the main chain and the further degradation of the residual structure, respectively. The peak at 332 °C represents the main weight loss rate, indicating that thermal cracking of the microcapsule wall network is most intense at this temperature range. Compared to the free core material, CBE-M exhibits significantly reduced weight loss at low temperatures, with the primary decomposition stage occurring at the temperature range at which the wall material undergoes major degradation. This suggests that the cross-linked capsule wall formed by recrystallisation of the gelatin–chitosan effectively inhibits the premature escape and degradation of the heat-sensitive active components in the core material. Consequently, the thermal stability of the cherry blossom fermentation extract is significantly improved [30,31,32,33,34,35,36].

#### 2.4.4. Analysis of the Sustained-Release Properties of Microcapsules

The results indicate that the release profile of the cherry blossom extract microcapsules exhibits a typical “two-phase” pattern overall, characterized by a rapid initial release followed by a gradual flattening in the later phase. This suggests that the gelatin–chitosan composite capsule wall exerts a significant retarding effect on the release process of the core material, thereby conferring the system with good sustained-release capabilities. This pattern is consistent with the overall research logic of this paper, which focuses on constructing dense, cross-linked capsule walls via the re-aggregation method to enhance the stability of active ingredients and slow down the release rate [29,30,31,32,33,34,35,36].

**Figure 11:**
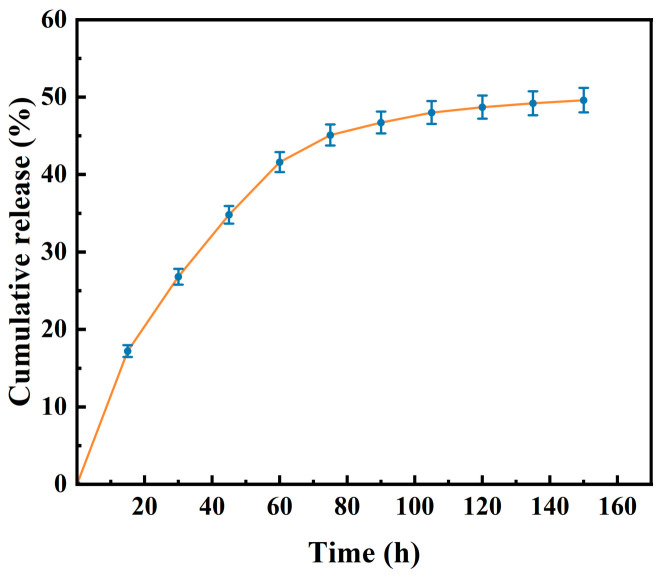
Sustained-release curve of microcapsules.

As shown in Figure 11, during the initial release phase (0–60 h), the cumulative release rate of the microcapsules rapidly increased from 0 to 41.6%, with a steep curve slope, indicating a distinct initial release stage in the system. This phenomenon is typically associated with two factors: First, during the recoagulation process, a portion of the core material is distributed on the capsule wall surface or in the shallow layer adjacent to the wall, allowing for preferential release under the influence of the external medium; second, the concentration gradient between the interior and exterior of the capsule is large during the initial release phase, making it easier for core molecules to migrate outward through the capsule wall network under the driving force of diffusion [29,30,31,32,33,34,35,36]. Therefore, the curve is steeper in the first half, reflecting the common “initial burst release” characteristic of recoagulation microcapsule systems.

When the release time extends beyond 75 h, the slope of the curve decreases significantly, and the release rate gradually slows down; within the 75–150 h range, the cumulative release rate increases only slowly from 45.1% to 49.6%, indicating that the system has entered a slow-release phase. At this point, the easily released surface core material had largely diffused out, and the remaining active ingredients were primarily embedded within the denser gelatin–chitosan cross-linked network. Their migration process was jointly constrained by diffusion resistance at the capsule wall, relaxation of polymer chains, and local swelling behavior; consequently, the overall release rate tended to stabilize. At 150 h, the cumulative release rate reached 49.6%, corresponding to an unreleased fraction of 50.4%. This result suggests that the gelatin–chitosan capsule wall could delay the diffusion of phenolic compounds from the microcapsules.

Combining the aforementioned characterization results from SEM, FTIR, and TGA, it can be concluded that the cross-linked capsule wall formed by the gelatin–chitosan co-precipitation method not only effectively encapsulates the core material structurally but also functionally endows the system with good sustained-release capacity and storage stability, thereby providing an important guarantee for the long-term efficacy of cherry blossom extract in antioxidant and antibacterial applications [29,30,31,32,33,34,35,36].

#### 2.4.5. Assessment of Antioxidant Activity

To systematically evaluate the retention of antioxidant activity in cherry blossom extract following microencapsulation and stabilization, and to further validate the effectiveness of the co-precipitation encapsulation technique in preserving bioactivity, this section focuses on unfermented cherry blossom extract (CBE-U), mixed-microbial fermented cherry blossom extract (CBE), and the release solution of microencapsulated mixed-microbial fermented extract (CBE-M) as the primary subjects of study. Additionally, the monomicrobial fermentation groups of *Monascus purpureus* (M-7 d) and *Bacillus subtilis* (B-7 d)—which exhibited the highest antioxidant activity in previous experiments—were introduced as cross-references. Three parameters—DPPH radical scavenging capacity, ABTS cation radical scavenging capacity, and total phenolic content (TPC)—were employed to evaluate the antioxidant performance of the samples following steady-state treatment [43,44,45].

**Figure 12:**
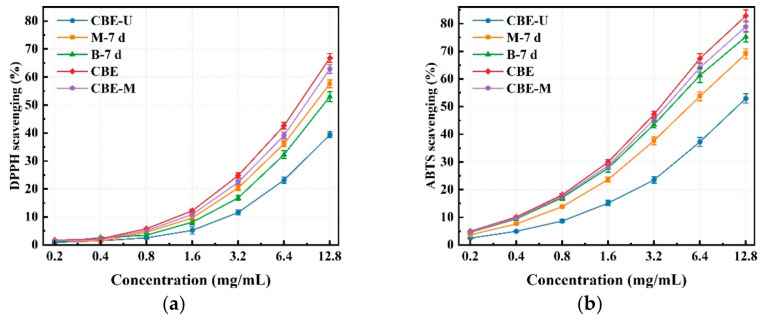
(**a**) DPPH radical scavenging capacity at different concentrations; (**b**) ABTS radical scavenging capacity at different concentrations.

The results indicate that after stabilization treatment, all samples still exhibited good in vitro antioxidant activity, with an overall trend of “mixed-microbial fermentation extract > microencapsulated release solution > optimal single-microbial fermentation group > unfermented extract” (Figure 12). In the DPPH system, the unfermented cherry blossom extract exhibited the lowest radical scavenging capacity, with a scavenging rate of 39.34% ± 1.06% at 12.8 mg/mL and an IC_50_ value of 18.62 ± 0.44 mg/mL as determined by fitting; at the same concentration, the optimal single-strain groups of *Monascus purpureus* and *Bacillus subtilis* increased to 57.55% ± 1.42% and 53.02% ± 1.76%, respectively, with IC_50_ values decreasing to 10.08 ± 0.27 mg/mL and 11.96 ± 0.31 mg/mL, respectively. This indicates that single-strain fermentation has significantly enhanced the sample’s ability to scavenge DPPH radicals. These results are consistent with those from previous single-strain fermentation experiments.

On this basis, the DPPH radical scavenging capacity of the mixed-microbial fermented cherry blossom extract was further enhanced, reaching 66.80% ± 1.62% at a concentration of 12.8 mg/mL, with the IC_50_ decreasing to 7.70 ± 0.22 mg/mL. The DPPH scavenging rate of the release solution from microencapsulated mixed-microbial fermentation extract was slightly lower than that of the free extract from mixed-microbial fermentation, but still reached 62.90% ± 1.54%, with an IC_50_ of 8.69 ± 0.24 mg/mL, and remained higher than that of the optimal groups from both single-microbial fermentation methods and the unfermented control across the entire concentration range. These results indicate that mixed-microbial fermentation can further promote the release of bound antioxidant components in cherry blossom tissue through enzymatic synergy. Although the DPPH scavenging capacity of the microencapsulated extract was slightly lower than that of the free extract due to the effects of encapsulation and the release process, the core antioxidant function was still largely preserved, suggesting that the co-precipitation encapsulation did not significantly weaken its antioxidant activity.

The results of ABTS cationic radical scavenging exhibited a pattern similar to that observed in the DPPH system, yet with certain differences. Unfermented cherry blossom extract demonstrated a certain scavenging capacity in the ABTS system, with a scavenging rate of 52.95% ± 1.68% at 12.8 mg/mL and an IC_50_ of 8.36 ± 0.21 mg/mL; the optimal single-strain groups of *Monascus purpureus* and *Bacillus subtilis* increased to 69.11% ± 1.79% and 75.30% ± 1.91%, respectively, with corresponding IC_50_ values of 5.02 ± 0.13 mg/mL and 4.06 ± 0.11 mg/mL.

Further comparison shows that the mixed-microbial fermentation extract exhibited the strongest antioxidant activity in the ABTS assay, with a scavenging rate of 82.80% ± 2.12% at a concentration of 12.8 mg/mL and a calculated IC_50_ of only 3.35 ± 0.10 mg/mL. The release solution of the microencapsulated mixed-microbial fermentation extract exhibited an ABTS scavenging rate of 78.90% ± 2.03% at the same concentration, with an IC_50_ of 3.72 ± 0.11 mg/mL, and outperformed the optimal single-microbial groups at all concentration points. Although the release solution from the microencapsulated extract was slightly lower than that of the unencapsulated mixed-microbial fermentation extract, it was still significantly higher than the optimal groups of both single-microbial fermentation extracts and the unfermented sample, indicating that the major antioxidant active components in the sample were well preserved during the release process following microencapsulation. Combining the DPPH and ABTS evaluation systems, it can be concluded that the microencapsulation process did not impair the free radical scavenging function of the mixed-microbial fermentation extract and endowed it with superior stability while maintaining a high level of antioxidant activity.

#### 2.4.6. Changes in Total Phenolic Content

**Figure 13:**
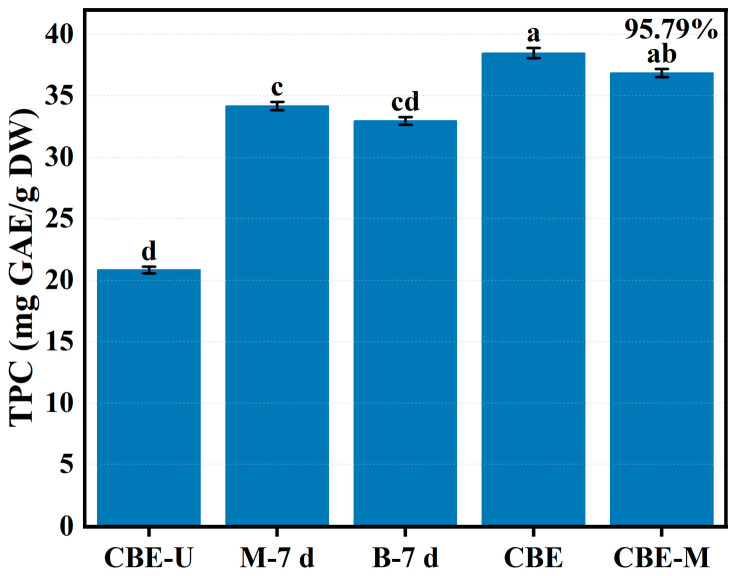
Total phenolic content. Different lowercase letters indicate significant differences between groups (*p* < 0.05).

The TPC results further support the above conclusions (Figure 13). The TPC of the unfermented cherry blossom extract was 20.84 ± 0.57 mg GAE/g DW; the optimal groups for *Monascus purpureus* and *Bacillus subtilis* single-strain fermentation increased to 34.18 ± 0.74 and 32.97 ± 0.71 mg GAE/g DW, respectively; the mixed-strain fermentation of cherry blossom extract yielded the highest value at 38.49 ± 0.41 mg GAE/g DW. The release solution from microencapsulated mixed-microbial fermentation extract was 36.87 ± 0.75 mg GAE/g DW, slightly lower than that of the free mixed-microbial extract but still at a high level. Overall, the trend in TPC changes was consistent with the changes in DPPH and ABTS scavenging capacities, indicating that total phenols remain the key component underlying the antioxidant capacity of this system. In terms of stabilization mechanisms, although the TPC of the release solution from the microencapsulated mixed-microbial fermentation extract was slightly lower than that of the free extract from the mixed microbial culture, the difference was negligible. Moreover, the DPPH and ABTS scavenging rates remained high, indicating that the gelatin–chitosan composite shell material effectively shielded the polyphenolic compounds in the core material from direct damage caused by oxygen, light, and the external environment to a certain extent.

Overall, this study provides preliminary evidence that microencapsulation can improve the stability and sustained release of fermented cherry blossom extract. However, because glutaraldehyde was used as the cross-linking agent, further optimization using non-toxic, food-grade, or cosmetically acceptable cross-linkers is required before practical application.

## 3. Materials and Methods

### 3.1. Materials

The plant substrate used in this experiment consists of petals from the ‘Kansan’ cherry tree. All petals were harvested during the peak blooming period in spring from mature cherry trees growing in a healthy ecological environment free of pesticide contamination. Immediately after harvesting, the petals are manually sorted in a clean environment to remove impurities, sepals, stamens, and any browned or damaged leaves. The processed fresh petals are spread evenly on stainless steel trays and first placed in a cool, well-ventilated room with controlled relative humidity for 24 h of natural air-drying. They are then transferred to a precision constant-temperature forced-air drying oven and gently dried at a constant low temperature of 40 °C until their weight stabilizes (typically taking 48 to 72 h). Low-temperature drying significantly minimizes the thermal degradation of heat-sensitive anthocyanins and volatile antioxidant compounds in the petals. The fully dried cherry blossom petals are sealed in light-proof vacuum bags and stored long-term at room temperature away from light, serving as standardized substrate material for subsequent fermentation experiments.

The strains of *Bacillus subtilis* and *Monascus purpureus* were both provided by the China General Microbial Culture Collection Center (CGMCC) and identified through molecular biological analysis.

### 3.2. Extraction of CBE

In this experiment, a 75% ethanol–water solution was selected as the extraction solvent. Cherry blossom petals were weighed and placed in a Soxhlet extractor. The ethanol solution was added according to the optimized solid-to-liquid ratio of 1:20 (g/mL). The Soxhlet extractor was securely fastened and placed in a water bath set at 80 °C, where it was heated to a gentle boil for reflux extraction. The extraction time was set to 4 h. After extraction, the mixture was allowed to stand and cool. The ethanol solvent was then removed from the extract under reduced pressure using a rotary evaporator (BÜCHI Labortechnik AG, Flawil, Switzerland). The resulting concentrate was freeze-dried to obtain concentrated cherry blossom extract (CBE), which was stored in a sealed container at 4 °C in the dark for subsequent analysis.

### 3.3. Mixed-Culture Solid-State Fermentation

*Monascus purpureus* and *Bacillus subtilis*, stored in glycerol vials at −80 °C, were revived on PDA and LB agar plates, respectively [22,28,37,38,39,40]. *Bacillus subtilis* was inoculated into LB broth and cultured at 180 rpm for 16–24 h. The viable cell density of *Bacillus subtilis* was determined by serial dilution and plate counting, and the suspension was adjusted to approximately 1.0 × 10^7^ CFU/mL using sterile saline. For *Monascus purpureus*, a spore suspension rather than a vegetative cell suspension was used as the fungal inoculum. Briefly, *Monascus purpureus* was cultured on PDA plates until sporulation, and spores were collected with sterile saline containing 0.05% Tween-80. The suspension was filtered through sterile gauze to remove mycelial fragments, and the spores were counted using a hemocytometer (Qiujing, Shanghai, China) and adjusted to approximately 1.0 × 10^7^ spores/mL.

Place 7.00 g of pretreated cherry blossom petals and 1.00 g of defatted rice flour in a 250 mL conical flask, add 4 mL of deionized water, and mix thoroughly to achieve an initial moisture content of approximately 50% in the substrate. Subsequently, sterilize at 121 °C and 0.1 MPa for 30 min, then cool to room temperature. Add a standardized bacterial suspension at a inoculation rate of 10% (*v*/*w*) of the initial dry weight of the substrate. For the mixed-culture group, inoculate with both bacterial suspensions according to the experimental design; for the control group, add an equal volume of sterile saline. The samples were subjected to solid-state fermentation at 30 °C. Under sterile conditions, the mixture was stirred once every 24 h for a total of 10 days, and samples were collected on days 5, 6, 7, 8, and 9.

For the unfermented control, the substrate was prepared using the same ratio of cherry blossom petals to defatted rice flour as that used in the fermented groups. This control was subjected to the same sterilization, incubation, and extraction procedures, except that an equal volume of sterile saline was added instead of microbial inoculum. Therefore, the unfermented control represented the same cherry blossom petal–rice flour substrate background without microbial fermentation.

To clarify the evaluation of factors affecting mixed-culture solid-state fermentation, three key variables were first investigated by single-factor experiments: total inoculation amount, inoculation proportion and fermentation time. For the inoculation amount assay, the *Bacillus subtilis*:*Monascus purpureus* ratio and fermentation time were fixed at 1:1 and 7 d, respectively, while the total inoculation amount was set at 6%, 8%, 10%, 12% and 14%. For the inoculation proportion assay, the total inoculation amount and fermentation time were fixed at 10% and 7 d, respectively, and *Bacillus subtilis*:*Monascus purpureus* ratios of 1:3, 1:2, 1:1, 2:1 and 3:1 were tested. For the fermentation time assay, the total inoculation amount and inoculation proportion were fixed at 10% and 1:1, respectively, and fermentation was performed for 5, 6, 7, 8 and 9 d. Based on the single-factor results, response surface methodology was further performed using a Box–Behnken design with inoculation amount (A: 8%, 10% and 12%), *Bacillus subtilis* volume fraction (B: 0.33, 0.50 and 0.67) and fermentation time (C: 6, 7 and 8 d) as independent variables, while TPC was used as the response variable and fitted to a second-order polynomial model using Design-Expert 13.0.

### 3.4. Preparation of Microcapsules

Microcapsules were prepared using a gelatin–chitosan co-precipitation method, with cherry blossom extract obtained through optimized mixed-microbial solid-state fermentation serving as the core material [30,31,32,33,34,35,36]. To prepare Solution A, weigh a specific amount of chitosan and dissolve it in a 1% glacial acetic acid solution. Then weigh out the required amount of gelatin (with a mass ratio of gelatin to chitosan of 10:1) and dissolve it in 100 mL of deionized water. Stir in a water bath at 50 °C until the gelatin has completely dissolved. Add the specified amount of cherry blossom extract (with a mass ratio of heartwood to pericarp of 1:1) and Tween 80 at a ratio of 2:1 to the solution. Sonicate at 700 W for five minutes until a homogeneous mixture is obtained (Solution B). Slowly add Solution A to Solution B over a period of 30 min, stirring continuously at 600 rpm and 50 °C to ensure the solution is thoroughly mixed. Adjust the pH of the mixture to 5.4 using a 10% sodium hydroxide solution and stir continuously at room temperature for one hour to allow the gelatin and chitosan to undergo reaggregation and form microcapsules. Once cooled to room temperature, stir the mixture in an ice-water bath for 30 min. Once the overall solution temperature has dropped below 5 °C, slowly add 0.5 mL of 25% glutaraldehyde to initiate the curing process. Continue stirring for four hours to form covalently cross-linked microcapsules. Filter, wash and dry the mixture to obtain CBE-M powder. Store the powder in a sealed container at 4 °C.

### 3.5. Determination of Total Phenolic Content

The total phenolic content (TPC) of the samples was determined using the Folin–Ciocalteu colorimetric method. Mix 0.05 mL of diluted sample with 0.25 mL of Folin–Ciocalteu solution and shake for 5 min; then, add 0.2 mL of 7.5% sodium bicarbonate solution. Allow the mixture to react in the dark at 25 °C for 1 h; then, measure the absorbance at 765 nm. Distilled water was used as the blank, and gallic acid as the standard. A standard curve was obtained: y = 0.0124x + 0.0087 (R^2^ = 0.9986). TPC was calculated using Equation (1), and the results are expressed in mg GAE/g DW [43]:

(2) $$TPC\;(mg\;GAE/g\;DW)=\frac{C\;\times V\;\times n}{m}$$

In the equation, *C* represents the concentration of the sample solution (mg/mL) calculated from the gallic acid standard curve; *V* represents the total volume of the sample extract (mL); *n* represents the dilution factor; and m represents the dry weight of the sample (g). All samples were analyzed in triplicate, and the results are expressed as the mean ± standard deviation.

### 3.6. DPPH Radical Scavenging Activity

The antioxidant activity of the samples was evaluated using the DPPH radical scavenging assay. A 1.0 × 10^−4^ mol/L DPPH ethanol solution was prepared and stored in the dark for later use. The samples were diluted with anhydrous ethanol to create a series of concentration gradients. For the experimental group, 0.10 mL of the sample solution was mixed with 0.10 mL of DPPH solution. After reacting in the dark for 1 h, the absorbance was measured at 517 nm and recorded as A_2_; for the control group, 0.10 mL of the sample solution was mixed with 0.10 mL of anhydrous ethanol, and the absorbance was measured and recorded as A_1_. For the blank group, 0.10 mL of DPPH solution was mixed with 0.10 mL of anhydrous ethanol, and the absorbance was measured and recorded as A_0_. The DPPH radical scavenging rate was calculated using Equation (3) [44]:

(3) $$DPPH\;scavenging\;activity\;(\%)=\left[1-\frac{A_{2\;}-{\;A}_{1}}{A_{0}}\right]\times 100$$

Here, A_0_ represents the absorbance of the blank group, A_1_ represents the absorbance of the sample control group, and A_2_ represents the absorbance of the experimental group. Dose–response curves were plotted based on the clearance rates at different concentrations, and the half-maximal clearance concentration (IC_50_) was calculated. All samples were tested in triplicate, and the results are presented as the mean ± standard deviation.

### 3.7. ABTS Radical Scavenging Activity

The antioxidant capacity of the samples was evaluated using the ABTS cation radical scavenging assay. A 7.4 mmol/L ABTS stock solution was mixed with a 2.6 mmol/L potassium persulfate stock solution in equal volumes and left at room temperature in the dark for 12–16 h to generate an ABTS+ radical working solution; we then diluted it with anhydrous ethanol until the absorbance at 734 nm was 0.70 ± 0.02. Prepare samples in anhydrous ethanol at different concentration gradients. For the experimental group, mix 50 μL of the sample solution with 450 μL of the ABTS working solution. After reacting in the dark for 20 min, measure the absorbance at 734 nm and record it as A2; for the control group, mix 50 μL of the sample solution with 450 μL of anhydrous ethanol, measure the absorbance, and record it as A1. For the blank group, 50 μL of anhydrous ethanol was mixed with 450 μL of ABTS working solution, and the absorbance was measured and recorded as A0. The ABTS radical scavenging rate was calculated using Equation (4) [45]:

(4) $$ABTS\;scavenging\;activity\;(\%)=\left[1-\frac{A_{2\;}-{\;A}_{1}}{A_{0}}\right]\times 100$$

Here, A_0_ represents the absorbance of the blank group, A_1_ represents the absorbance of the sample control group, and A_2_ represents the absorbance of the experimental group. Dose–response curves were plotted based on the clearance rates at different concentrations, and the IC_50_ values were calculated. All samples were tested in triplicate, and the results are expressed as the mean ± standard deviation.

### 3.8. Observation by Scanning Electron Microscopy

Dried cherry blossom extract microcapsules were placed on the surface of a copper sample holder and gold-sputtered; the structural characteristics of the microcapsules were then observed using a scanning electron microscope (Hitachi High-Tech Corporation, Tokyo, Japan).

### 3.9. Analysis of Fourier Transform Infrared Spectroscopy

Fourier transform infrared spectroscopy (FTIR)(Thermo Fisher Scientific, Waltham, MA, USA) was used to analyze cherry blossom extract, gelatin, chitosan, and microcapsules containing cherry blossom extract. Solid powders were pressed into KBr pellets, and each sample was scanned 16 times in the wavenumber range of 500–4000 cm^−1^ at a resolution of 4 cm^−1^. The measured IR spectra were processed and analyzed using analytical software (OriginPro 2023).

### 3.10. Analysis of the Thermal Stability of Microcapsules

Thermogravimetric analysis (TGA) (TA Instruments, New Castle, DE, USA) was performed to determine the thermogravimetric curves of gelatin, chitosan, cherry blossom extract microcapsule powder, and cherry blossom extract, respectively. Weigh 8 mg each of CBE, gelatin, chitosan, and CBE-M and place them into the TGA furnace. The temperature was raised from 0 °C to 600 °C at a constant rate of 10 °C/min, using nitrogen as the protective gas at a flow rate of 20 mL/min. The measured thermogravimetric curves were processed and analyzed using analytical software (OriginPro 2023).

### 3.11. Standard Curve for Cherry Blossom Extract

**Figure 14:**
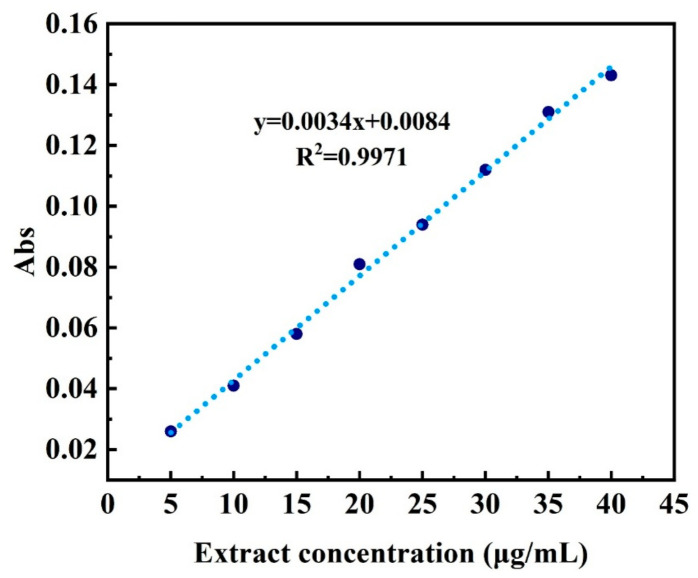
Standard curve for CBE.

An ethanol solution of cherry blossom extract was scanned over the wavelength range of 200–800 nm to determine its maximum absorption wavelength. The maximum absorption wavelength was determined to be 325 nm. The absorbance of various diluted ethanol solutions was measured at 325 nm to establish the relationship between absorbance and the concentration of the cherry blossom extract, and a standard curve for the essential oil of the cherry blossom extract was plotted. The results (Figure 14) showed that within the range of 5–40 μg/mL, the absorbance exhibited a good linear relationship with concentration. The regression equation was y = 0.0034x + 0.0084, with R^2^ = 0.9971.

### 3.12. Analysis of the Sustained-Release Properties of Microcapsules

First, accurately weigh 10 g of dried cherry blossom extract microcapsules and place them in a glass Petri dish, and then store them at a constant temperature of 25 °C. Every 15 h, weigh a portion of the microcapsules, soak them in 50 mL of anhydrous ethanol for 1 h, then subject them to intermittent ultrasonication (Ningbo Scientz Biotechnology Co., Ltd., Ningbo, China) at 700 W for 30 min to break down the microcapsules. Next, centrifuge at 8000 rpm for 5 min, collect the supernatant, dilute it, and measure its absorbance under UV light. The relative cumulative release rate of the microcapsules was calculated using the following formula to evaluate their sustained-release performance.

(5) $$Cumulative\;release\;\left(\%\right)=\left[\frac{A_{0}-A_{1}}{A_{0}}\right]\times 100$$

Here, A_0_ is the initial extract concentration, and A_1_ is the extract concentration at the time of sampling.

### 3.13. Determination of Key Degradative Enzyme Activity During Fermentation

(1) Extraction of crude enzyme solution: Lactate–sodium lactate buffer (0.05 mol/L, pH 3.0), acetic acid–sodium acetate buffer (0.05 mol/L, pH 4.0), disodium hydrogen phosphate–citric acid buffer (0.2 mol/L, pH 6.0), phosphate buffer (0.2 mol/L, pH 7.0, pH 7.5), and boric acid buffer (0.05 mol/L, pH 10.5) were used to extract different enzymes. A specific amount of fermented cherry blossom petals and control samples was taken and added to different buffers at a substrate-to-buffer ratio of 1:10 (*w*/*v*). The mixture was placed in a constant-temperature shaker at 180 rpm and 30 °C for 1 h of extraction. Subsequently, the mixture was centrifuged at 4 °C and 10,000 rpm for 20 min, and the supernatant was collected as the crude enzyme solution. After extraction, the crude enzyme solution was stored at −20 °C for subsequent analysis.
(2) Endoglucanase activity assay: 100 μL of 1% sodium carboxymethyl cellulose solution was heated at 50 °C for 10 min; 50 μL of the crude enzyme solution was added, and the mixture was heated at 50 °C for 30 min. Immediately thereafter, 150 μL of DNS was added and reacted in boiling water for 5 min. After cooling to room temperature, add 700 μL of water and measure the absorbance at 540 nm.
(3) Exoglucanase activity assay: Place a 0.5 × 2 cm filter paper in a test tube, add 100 μL of 50 mM sodium citrate buffer (pH 5.0), heat at 37 °C for 10 min, then add 50 μL of crude enzyme solution, heat at 37 °C for 60 min, and immediately add 150 μL of DNS, and then react in boiling water for 5 min. After cooling to room temperature, add 700 μL of water and measure the absorbance at 540 nm. The difference in the control group is that the DNS solution is added first, followed by the crude enzyme solution.
(4) Xylanase activity assay: Heat 100 μL of 1% xylan solution at 50 °C for 5 min, add 50 μL of crude enzyme solution, heat at 50 °C for 30 min, then immediately add 150 μL of DNS, react in boiling water for 5 min, cool to room temperature, add 700 μL of water, and measure the absorbance at 540 nm.
(5) Lignin peroxidase activity assay: Mix 40 μL of 1 mM resveratrol solution, 140 μL of crude enzyme solution, and 20 μL of 0.2 mM H_2_O_2_ solution; incubate at 37 °C for 3 min, then measure the absorbance at 310 nm. For the control group, replace the H_2_O_2_ solution with distilled water.

The actual number of moles of reducing sugars released during the enzymatic reaction is calculated using standard calibration curves (with a coefficient of determination R^2^ > 0.999) plotted in advance by reacting a series of anhydrous glucose standards (for calculating cellulase activity) and D-xylose standards (for calculating xylanase activity) with DNS reagent to produce a color reaction. The unit of activity (U) for a single cellulase or xylanase is defined as the mass of enzyme protein required to continuously catalyze the hydrolysis of a specific substrate to release 1 μmol of reducing sugar (glucose equivalent or xylose equivalent) per minute (min) under the optimal pH and reaction temperature conditions set above. The final test results are converted and uniformly expressed in U/g dry weight (absolute enzyme activity per gram of dry weight of the fermented cherry blossom substrate).

## 4. Conclusions

This study demonstrates that solid-state co-fermentation of *Bacillus subtilis* and *Monascus purpureus* can effectively enhance the release of phenolic compounds and the antioxidant activity of cherry blossom extract. Based on response surface optimization, the optimal fermentation conditions were an inoculation amount of 10.5%, a *Bacillus subtilis* to *Monascus purpureus* inoculation proportion of 1.3:1, and a fermentation duration of 7.3 days; under these conditions, the total phenolic content of the sample reached 38.49 ± 0.41 mg GAE/g DW. Combined with the results of previous single-strain fermentation, it is evident that the two strains exhibit significant functional complementarity in their enzymatic systems: *Monascus purpureus* is more effective at degrading the cellulose backbone and releasing hydrogen-donating phenolics, while *Bacillus subtilis* is more advantageous in breaking down hemicellulose and lignin barriers; therefore, the mixed-strain system can further promote the release of bound phenolics and enhance free radical scavenging capacity.

Based on these findings, the gelatin–chitosan co-precipitation method successfully achieved the microencapsulation of the optimal mixed-microbial fermentation extract. During in vitro release, the microcapsules exhibited a typical “fast-then-slow” profile: they entered a slow-release phase after 75 h, with a cumulative release rate of 49.6% at 150 h, leaving an unreleased fraction of 50.4%, indicating that this capsule wall system effectively delays the escape of active ingredients and improves system stability. Meanwhile, although the apparent antioxidant capacity of the release solution from the microcapsules was slightly lower than that of the free extract from the mixed-microbial fermentation, it was still significantly superior to that of the optimal single-microbial fermentation group and the unfermented extract. This indicates that the encapsulation process did not significantly weaken the core antioxidant function but rather facilitated the sustained release and functional retention of the active components. Overall, this study has established a comprehensive technical framework ranging from “synergistic mixed-culture fermentation” to “microcapsule stabilization,” providing an experimental basis for the development and application of active cherry blossom extracts in natural antioxidants, functional food ingredients, and related bioactive materials.

## Figures and Tables

**Table 1:** Box–Behnken experimental factors and levels.

Level	A Inoculation Amount (%)	B Inoculation Proportion ^1^	C Fermentation Time (d)
−1	8	0.33	6
0	10	0.50	7
1	12	0.67	8

^1^ Inoculation proportions of 0.33, 0.50 and 0.67 correspond to *Bacillus subtilis* and *Monascus purpureus* ratios of 1:2, 1:1 and 2:1 respectively.

**Table 2:** Box–Behnken response surface design and results.

Run	A Inoculation Amount (%)	B Inoculation Proportion ^1^	C Fermentation Time (d)	TPC (mg GAE/g DW) ^1^
1	8	0.33	7	34.20 ± 0.42
2	12	0.33	7	34.50 ± 0.45
3	8	0.67	7	34.75 ± 0.44
4	12	0.67	7	37.26 ± 0.51
5	8	0.50	6	31.97 ± 0.38
6	12	0.50	6	33.36 ± 0.45
7	8	0.50	8	34.50 ± 0.42
8	12	0.50	8	36.51 ± 0.51
9	10	0.33	6	32.97 ± 0.41
10	10	0.67	6	34.01 ± 0.45
11	10	0.33	8	35.91 ± 0.46
12	10	0.67	8	36.53 ± 0.50
13	10	0.50	7	37.84 ± 0.47
14	10	0.50	7	37.87 ± 0.54
15	10	0.50	7	38.43 ± 0.55
16	10	0.50	7	38.32 ± 0.52
17	10	0.50	7	38.54 ± 0.49

^1^ The mean values ± standard deviation (*n* = 3).

**Table 3:** Results of the analysis of variance for the response surface model.

Source	Sum of Squares	DF	Mean Square	F-Value	*p*-Value
Model	70.3937	9	7.8215	67.49	<0.0001
A	4.8205	1	4.8205	41.59	0.00035
B	3.0876	1	3.0876	26.64	0.00131
C	15.5124	1	15.5124	133.85	<0.0001
AB	1.2210	1	1.2210	10.54	0.0141
AC	0.0961	1	0.0961	0.83	0.3928
BC	0.0441	1	0.0441	0.38	0.5569
A^2^	15.1401	1	15.1401	130.63	<0.0001
B^2^	5.3408	1	5.3408	46.08	0.00026
C^2^	20.7278	1	20.7278	178.85	<0.0001
Residual	0.8113	7	0.1159		
Lack of Fit	0.3899	3	0.1300	1.23	0.4073
Pure Error	0.4214	4	0.1054		
Total	71.2050	16			

## Data Availability

Data are contained within the article.

## Abbreviations

The following abbreviations are used in this manuscript:

CBE	Cherry Blossom Extract
TPC	Total Phenolic Content
BHT	Butylated Hydroxytoluene
BHA	Butylated Hydroxyanisole
PDA	Potato Dextrose Agar
LB	Luria–Bertani
CBE-U	Unfermented Cherry Blossom Extract
CBE-M	Cherry Blossom Extract Microcapsules
M-7 d	Cherry blossom extract fermented with *Monascus purpureus* for 7 days
B-7 d	Cherry blossom extract fermented with *Bacillus subtilis* for 7 days
TG	Thermogravimetry
DTG	Derivative Thermogravimetry
DPPH	1,1-Diphenyl-2-picrylhydrazyl
ABTS	2,2′-azino-bis(3-ethylbenzothiazoline-6-sulfonic acid) radical cation
